# The Best Interest of the Patient

**DOI:** 10.1177/1062860620940290

**Published:** 2020-07-16

**Authors:** David J. Ballard, David B. Nash

**Affiliations:** 1 University of North Carolina at Chapel Hill, NC; 2 Jefferson College of Population Health, PA

Vice President Michael Pence’s choice to not wear a mask while visiting Mayo Clinic on April 28, 2020, and Mayo Clinic’s decision to allow this to occur in what was known to be a very public event illustrates important opportunities and lessons for global public health, as well as health care system executive leadership and patient safety across health care systems.

As one of us (DJB) is a Mayo Clinic internal medicine training program alumnus, former Mayo Clinic Consultant in the 1980s, and Healthcare Section Policy Editor for the *Mayo Clinic Proceedings*, and the other (DBN) has worked more than 3 decades on issues pertaining to public accountability of health care systems, physician leadership, and quality of care, while often citing the Mayo Clinic as an exemplar in these arenas, we were stunned on April 28 with the news from Rochester.

The video images of the Vice President of the United States and Chair of the COVID-19 Task Force, without a face mask, elbow bumping with a Mayo patient, whereas the entourage surrounding him all donned face masks, were deeply disappointing. Although Pence later stated on May 3, “I should have worn a mask at the Mayo Clinic,”^1^ and recognizing that Mayo Clinic has renewed its commitment to its face mask policy since this event through messaging that Mayo Clinic is “Your safe destination for face-to-face care,”^2^ our immediate reaction was, “If this patient safety policy violation can occur at the Mayo Clinic, it can happen anywhere in the world.”

The Mayo Clinic’s commitment to patients and to patient safety is long-standing and legendary. Mayo Clinic’s core value—“The needs of the patient come first”—described more than a decade ago evolves from William J. Mayo’s articulation that “the best interest of the patient is the only interest to be considered” more than a century ago in a commencement address to the Rush Medical School graduating class in 1910.^3,4^ We have admired Mayo Clinic’s deep commitment to patient safety culture and to policies and practices that support reducing health care harm. With this backdrop, Mayo Clinic’s failure to intervene to avoid the violation of its face mask policy on April 28, particularly in Mayo clinical care areas, was deeply disappointing and presents important lessons in executive leadership and patient safety for health care organizations globally.

As articulated during our interview with him related to this event and as discussed in more detail in his book on health care leadership,^5^ Denis Cortese, former Chief Executive Officer of Mayo Clinic and the original chair of the Roundtable on Value and Science-Driven Health Care of the National Academy of Medicine, stated,

The role of senior leadership in any organization is to create the vision; communicate messages in a way that everyone begins to take ownership; and align the organization for success in reaching the goals. In the case of Mayo Clinic, the reason to exist is to care for patients. The “needs of the patients come first” is a core value that every Mayo Clinic employee believes and owns. Senior leaders must have the courage, will, and vision to consistently reinforce the message. Leaders should do so through actions, not just words. The responsibility for maintaining the core value lies with Mayo internal leadership. The responsibility of the Board of Trustees is to understand, endorse, and ensure this message is reinforced. To me, if Vice President Pence was unwilling to wear a mask, his visit on the Mayo campus should have been terminated on the spot and moved off the campus. This would have reinforced the message of the Mayo Clinic’s core value, rather than cause a disconnect between what the Mayo Clinic staff live and breathe every day and the actions of its senior leadership. The Mayo Clinic culture is strong, but not immune to change if leadership does not reinforce it in visible ways.

Mayo Clinic is the world’s leading brand in health care and an organization with a world-renowned commitment to patient safety. A forthcoming commentary in the *American Journal of Medical Quality* focuses on how this patient safety policy violation event might be used as a learning experience to advance health care leadership and patient safety globally. Mayer and Hatlie address the executive leadership and patient safety lessons learned from a tragic patient safety event in another health care organization.^6^ Additionally, they provide cogent recommendations for how health care systems can learn from this episode to optimize a global commitment to patient safety and to the reliable performance of patient safety practices.

To reinforce their conclusions, health care systems need to focus on 3 components to achieve and sustain a commitment to patient safety culture and to reliably implement patient safety practices: an engaged and courageous leadership that walks the walk, transparency that fuels learning and improvement from all safety events, and true partnerships with patients and families so that health care systems throughout the world can embody the Mayo core value that “the needs of the patient come first.”
